# KRAB family is involved in network shifts in response to osmotic stress in camels

**DOI:** 10.1080/19768354.2022.2143894

**Published:** 2022-11-11

**Authors:** Dandan Cao, Shenyuan Wang, Dong Zhang, Yanru Zhang, Junwei Cao, Yongbin Liu, Huanmin Zhou

**Affiliations:** a College of Life Science, Inner Mongolia Agricultural University, Hohhot, People’s Republic of China; b Sheep Collaboration and Innovation Center, Inner Mongolia University, Hohhot, People’s Republic of China

**Keywords:** KRAB family, osmotic stress, transcriptional networks, camels

## Abstract

A feature of the camel is its tolerance to osmotic stress. However, few studies of osmotic stress in vivo or comparative analyses between different tissues of the camel have been performed. Here, we report the roles of Krüppel-associated box domain containing zinc-finger repressor proteins (KRAB-ZFPs) in transcriptional networks under osmotic stress in camels by analyzing transcriptomes of four different tissues under various osmotic conditions. We found that 273 of 278 KRAB-ZFPs were expressed in our data set, being involved in all of the 65 identified networks and exhibiting their extensive functional diversity. We also found that 110 KRAB-ZFPs were hub genes involved in more than half of the networks. We demonstrated that the osmotic stress response is involved in network shifts and that KRAB-ZFPs mediate this process. Finally, we presented the diverse mechanisms of osmotic stress responses in different tissues. These results revealed the genetic architecture of systematic physiological response in vivo to osmotic stress in camels. Our work will lead to new directions for studying the mechanism of osmotic stress response in anti-arid mammals.

## Introduction

Hyperosmotic stress occurs when the osmotic equilibrium between extracellular and intracellular compartments is disrupted. Many studies have implicated hyperosmotic stress in tissue-specific disorders (Schilli et al., 1982, Vernia et al., 1988, Neuhofer, 2010, Pan et al., 2011). Hyperosmotic stress can trigger a wide range of detrimental cellular and molecular events such as cell shrinkage, oxidative stress, protein carbonylation, mitochondrial depolarization, DNA damage, and cell cycle arrest, leading to cell apoptosis (Brocker et al., 2012). To avoid these adverse impacts, mammalian tissues and cells have evolved many adaptive response mechanisms to restore the osmotic equilibrium. Recently, a growing number of studies indicate that numerous genes, not just one, are involved in the response to hyperosmotic stress in mammalian tissues. To date, the analyses have focused on individual gene-transcript changes when animals are exposed to osmotic stress, but these studies have not revealed the crucial switching of gene networks during the process of osmotic stress response. As a result, the answers to two questions remain elusive: (1) What are the key genes that mediate the shift among different transcriptional networks? (2) Do different tissues exhibit tissue-specific patterns in response to osmotic stress? A major reason for the former may be the neglect of the Krüppel-associated box domain containing zinc-finger repressor proteins (KRAB-ZFPs), which arose in tetrapods and have been dramatically amplified in mammals (Bellefroid et al. 1995; Looman et al. 2002; Hamilton et al. 2003), e.g. ∼400 copies in humans (Huntley et al., 2006). Furthermore, a recent study has indicated that most KRAB proteins bind to DNA regulatory regions in the human genome, implying their broad range of regulatory targets (Najafabadi et al., 2015). In our experiments on camel osmotic stress, we found that the KRAB-ZFP coding genes were broadly expressed in camel tissues during the analysis of differentially expressed gene data. Emerging evidence suggests that KRAB-ZFPs regulate multiple biological processes such as stem cell pluripotency, early embryonic development and differentiation, genomic imprinting, response to DNA damage, and behavioral stress.

With the above motivation, we hypothesized that KRAB-ZFPs are involved in the transcriptional network shift under hyperosmotic stress, allowing cells to restore osmotic equilibrium to avoid adverse effects. To test this hypothesis, we assessed: (1) whether KRAB-ZFPs have the potential to regulate substantial numbers of genes in camels; (2) whether KRAB-ZFPs are key regulators in the networks; and (3) whether a network shift occurs when tissues are under osmotic stress. We sequenced mRNA from the renal cortex, renal medulla, colon, and adrenal gland of five domesticated camels under varied osmotic conditions (salt restriction (SR), water restriction (WR), salt stress (SS), guzzling after water restriction (GWR), and control) and performed a comprehensive analysis. Understanding the process of transcriptional network shift under osmotic stress could provide insights into the mechanism of response to osmotic stress or the disorders caused by hyperosmotic stress.

## Results and discussion

### Physical condition supervision

**Figure 1.**
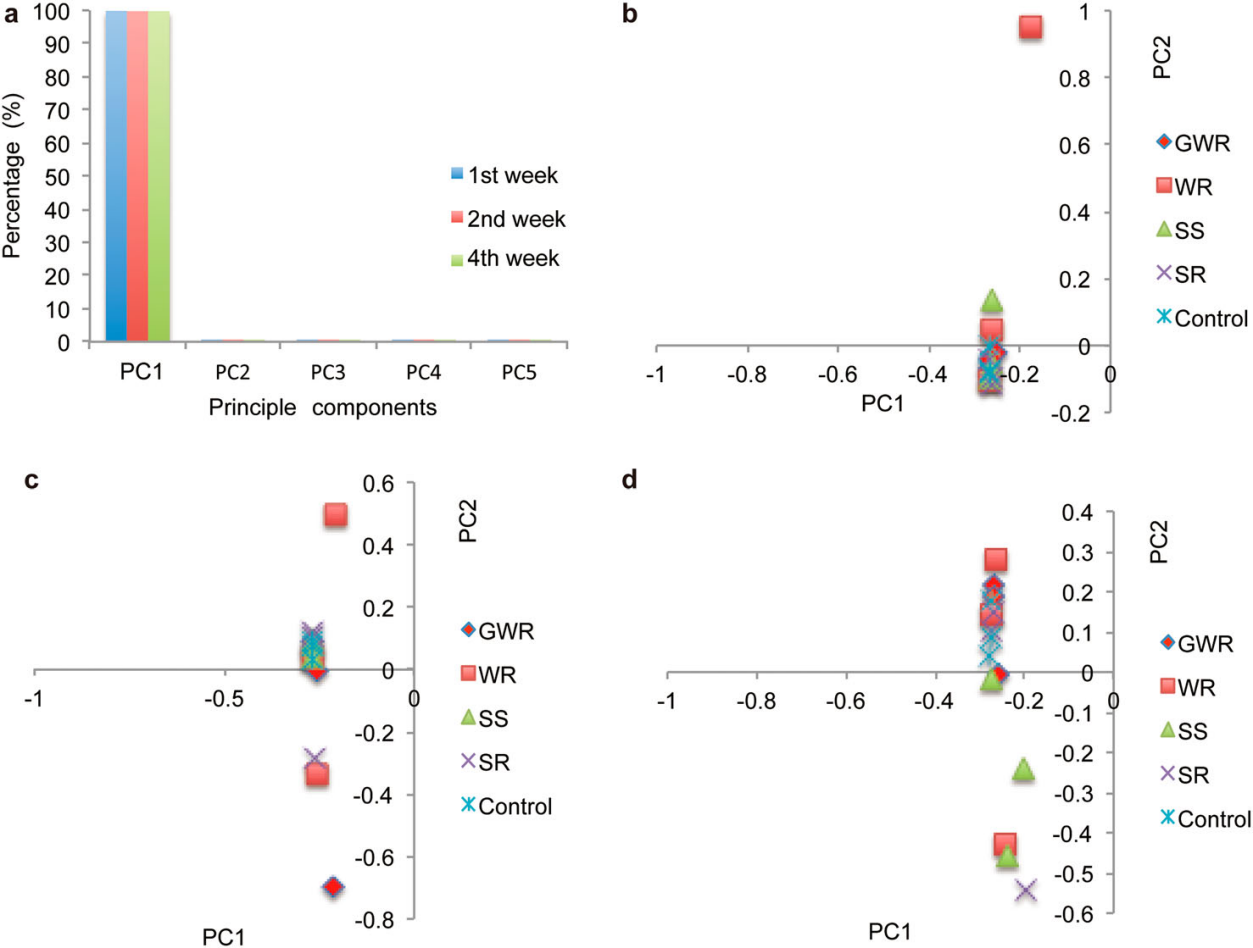
PCA analysis for the blood samples collected for physical condition supervision. (a) Contribution of the first five principle components (PC) for explaining the variability of the blood samples. PCA analyses were performed on the blood datasets of the 1st, 2nd, and 4th week. The result of 1st week is shown in blue, 2nd week red and 4th week green. (b) Results obtaining from PCA using blood data of the 1st week. PC1 and PC2 were shown. (c) Results obtaining from PCA using blood data of the 2nd week. (d) Results obtaining from PCA using blood data of the 1st week. Clearly, PC1 can't separate the samples from different groups.

For further comparative transcriptomic analysis, we first investigated whether the experimental camels were in healthy physiological condition, as this was indispensable for deriving an appropriate explanation of the mechanism of hyperosmotic stress response. Then, we carried out principal component analysis (PCA) (Patterson et al., 2006) on the physiological data of blood and urine measured under different osmotic stressors and for different periods. The results showed that the first eigenvector could explain over 99% of the variance in blood samples in all three sets of physiological data derived from blood (99.85%, 99.77%, and 99.76% for the first, second, and fourth weeks, respectively) (Supplementary Table 6 and Figure 1a). In addition, it could not separate the experimental individuals (Figure 1b, c, d), suggesting that homeostasis of blood was well maintained regardless of treatments and treatment time, consistent with the camel's remarkable hyperosmotic stress tolerance. However, the proportion of the variance in the urine examination data sets explained by the first eigenvector varied markedly (58.97%, 35.28%, and 42.15% for the first, second, and fourth weeks, respectively) (Supplementary Tables 7 and Supplementary Fig. 1). In addition, in the first week the first eigenvector separated the control group from others (Supplementary Fig. 2). The observations suggested that metabolic components in the urine under different treatments were distinct. The results met our expectations because camels needed to change the metabolic networks to maintain homeostasis in their body under osmotic stress conditions. Our analysis demonstrated that experimental camels were still healthy after a long period of osmotic stress.

Exposure to osmotic stressors could lead to abnormal tissues or cells in camels. As reported previously, hyperosmotic stress can trigger a wide range of cellular and molecular processes such as cell shrinkage, oxidative stress, and cell apoptosis (Brocker et al., 2012). In our case, we assessed the health status based on PCA derived from physiological data (Supplementary Table 6 and Figure 1a) using routine biochemical indices generated from analyses of the blood and urine (Supplementary Tables 2–4). In contrast, the physiological and biochemical components in blood in the treated groups were identical to those of the controls. These results implied that the camels retained normal physiological conditions. However, the metabolic components were varied in the urine at different times, by which it was inferred that camel tissues activated response networks to the osmotic stressors for maintaining homeostasis.

### Identification and overview of KRAB-ZFPs in camels

**Figure 2.**
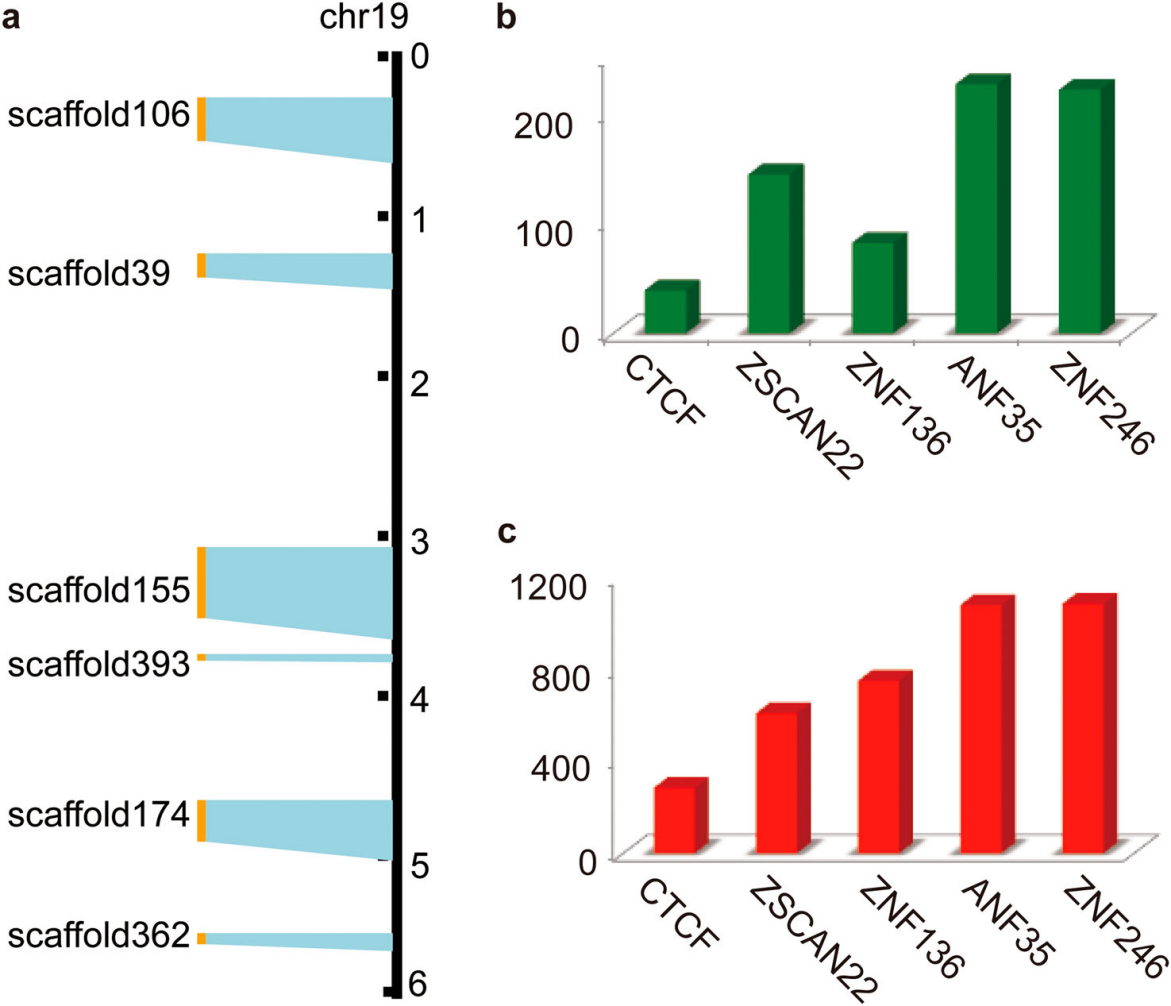
Overview of KRAB-ZFP genes and their potential regulatory genes in Bactrian camels. (a) Colinearity between camel's scaffolds containing tandem KRAB-ZFP genes and human's chromosome 19, which contains majority of KRAB-ZFP genes. (b) Statistics of genes regulated by CTCF, ZSCAN22, ZNF136, ANF35 and ZNF246 genes when the exerting distance is set to 1000 bp. (c) Statistics of genes regulated by CTCF, ZSCAN22, ZNF136, ANF35 and ZNF246 genes when the exerting distance is set to 100 kb.

**Figure 3.**
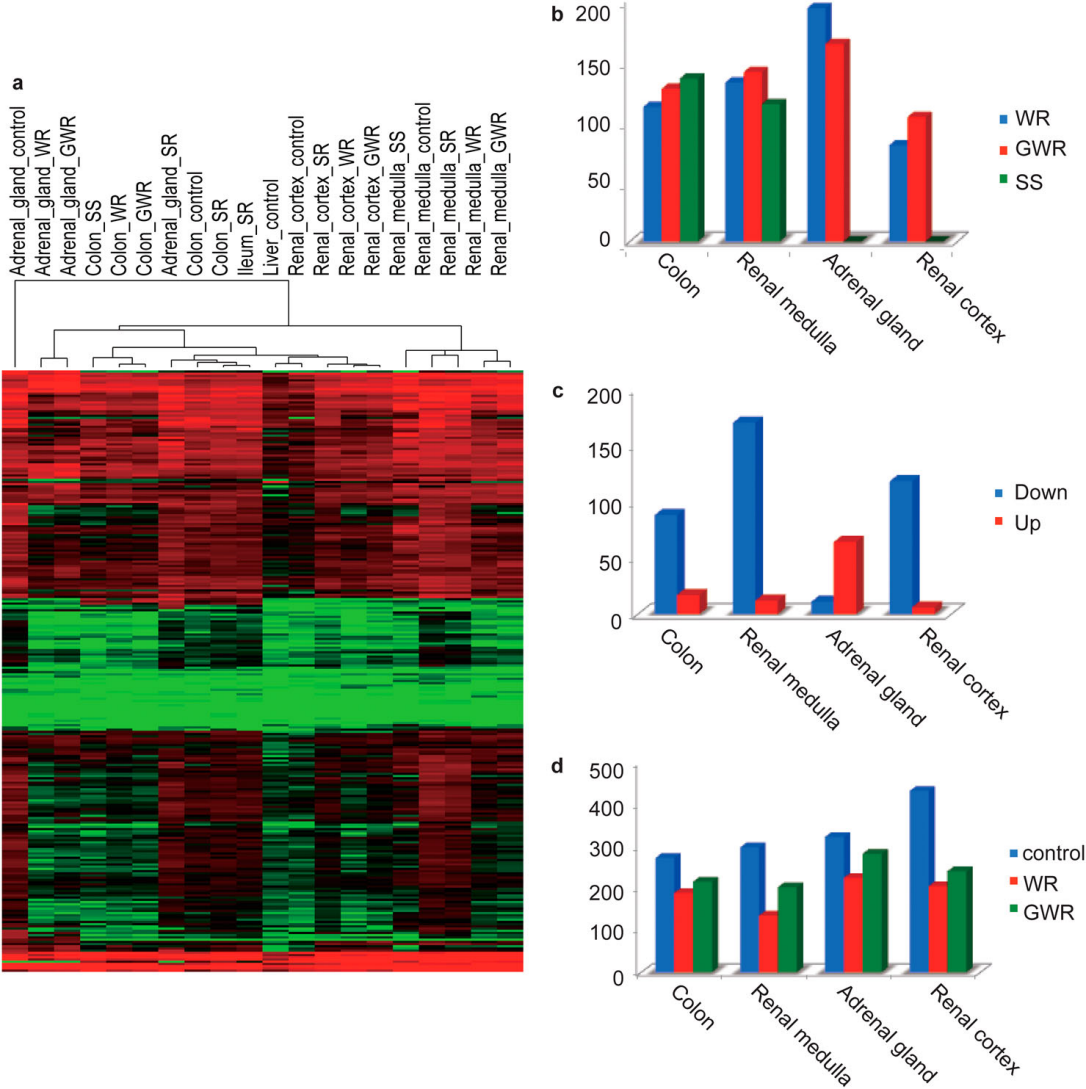
Expression overview of KRAB-ZFP genes under different treatments. (a) Expression of all the expressed KRAB-ZFP genes in each sample using heatmap. The redder the color is, the higher the expression is. The greener the color is, the lower the expression is. (b) Statistics of differentially expressed KRAB-ZFP genes under WR, GWR and SS conditions within tissues compared with control. WR treatment is denoted with blue, GWR red and SS green. (c) Statistics of differentially downregulated and upregulated KRAB-ZFP genes under WR condition in colon, adrenal gland, renal cortex and renal medulla. Downregulated genes are indicated as blue, upregulated genes red. (d) Statistics of alterative splicing events occurring in KRAB-ZFP genes in Control, WR and GWR groups.

We investigated the expression profiles under varied levels of osmotic stress in camel tissues and found that the majority (∼90%) of the KRAB-ZFP coding genes were expressed in each sample (Supplementary Table 9) based on differentially expressed genes, reflecting their broad expression (Figure 3a). Functional annotation of Bactrian camel genes (n = 20,251) based on the Kyoto Encyclopedia of Genes and Genomes (KEGG) database (Kanehisa & Goto, 2000) showed that the Bactrian camel genome has 278 copies of KRAB-ZFP coding genes (KO number: K09228), accounting for 1.4% of the total number of genes, ranking second after olfactory receptors (n = 735, K04257). The number is much higher than the third (n = 67, protein-tyrosine phosphatase, K01104) and fourth most numerous genes (n = 51, ankyrin, K10380) (Supplementary Table 8). Visualization of these KRAB-ZFP coding genes on scaffolds showed that approximately 81% of genes (n = 225) were tandem distributed on the scaffolds and had collinearity with the human genome (Supplementary Fig. 3). Interestingly, six scaffolds containing tandem duplicated KRAB-ZFP coding genes were well aligned to the six fragments of human chromosome 19 (Figure 2a) that have been indicated as possessing the most KRAB-ZFP coding genes in humans (Lukic et al., 2014). This was consistent with the notion that KRAB-ZFP coding genes have experienced considerable expansion in mammals. However, we observed fewer KRAB-ZFP coding genes in camels compared with humans (278 vs ∼400). One plausible explanation may be that primates experienced another round of amplification when endogenous retrovirus families invaded the genomes after speciation (Jacobs et al., 2014).

KRAB-ZFPs negatively regulate target genes by binding to their regulatory regions. They have been found to bind to a wide range of DNA regulatory regions in humans, suggesting that their regulative targets may be involved in a diverse range of genes and networks (Najafabadi et al., 2015). To assess whether KRAB-ZFPs could also regulate multiple genes in camels, we searched the recently identified regulatory regions of five KRAB-ZFPs (ZSCAN22, CTCF, ANF35, ZNF264, and ZNF136) (Najafabadi et al., 2015) in the camel genome using BLAST (Altschul et al., 1990). In total, 2,461 candidate regulatory regions were identified. We then asked how many genes might be regulated. Since regulatory regions are commonly located within a few kilobases (kb) of their target genes, we arbitrarily defined ‘a regulated gene’ if it was located one kilobase downstream or upstream of a given regulatory region. With this criterion, 712 candidate genes were identified (Figure 2b). Specifically, previous studies demonstrated that many regulatory regions could exert their influence over a distance greater than 100 kb (Mifsud et al., 2015). When we set the criterion to 100 kb, we identified 3,810 candidates, an average of ∼700 target genes for each KRAB-ZFP (Figure 2c). Taken together, we believe the results suggest that KRAB-ZFPs may also regulate a large number of genes and networks in camels.

### Expression of KRAB-ZFPs under varied osmotic stress conditions in camels

Although the above section theoretically provides evidence for multiple regulatory targets of KRAB-ZFPs, the role of KRAB-ZFPs in osmotic stress response needed further investigation. Our transcriptome results indicated that 90% of the KRAB-ZFP coding genes were expressed in each sample (Supplementary Table 9 and Figure 3a). For each tissue, we analyzed differential expression patterns (upregulation: fold change ratio ≥ 2, FDR <0.01; downregulation: fold change ratio ≤ 2, FDR < 0.01) between WR and CG, GWR and CG, and SS and CG followed by KO category enrichment. The enrichment results showed that under WR, SS, and GWR treatments, KRAB-ZFP (K09228) was highly enriched in the considered tissues (all *p* < 0.001, hypergeometric test; Supplementary Table 10). Indeed, the number of differentially expressed genes associated with K09228 was the highest among all KO categories in each tissue. As indicated by our results, under WR conditions, 105, 182, 124, and 75 KRAB-ZFP coding genes were differentially expressed in the colon, adrenal gland, renal medulla, and renal cortex, respectively (Figure 3b), corresponding to 119, 154, 132, and 97 KRAB-ZFP coding genes under GWR conditions (Figure 3b). Salt stress led to 127 and 107 differentially expressed KRAB-ZFP coding genes in the colon and renal medulla (Figure 3b). It is noteworthy that among these differentially expressed genes, over 90% were downregulated in all tissues, except for the renal cortex (Figure 3c; Supplementary Figs. 4 and 5). Since most KRAB-ZFPs are repressors, their downregulation will result in the activation of their target genes or networks. The observation of a large number of differentially expressed KRAB-ZFP genes under different osmotic stresses in these tissues may suggest the important role of KRAB-ZFPs in osmotic stress response.

Earlier studies demonstrated that alterative splicing events (ASEs) could occur in KRAB-ZFP coding genes, yielding more KRAB-ZFP isoforms and thereby broadening their functional diversity (Rosati et al., 1999; Resch et al., 2004). Hence, we next evaluated the changes in alterative splicing events in KRAB-ZFPs. Overall, ASEs existed in KRAB-ZFP genes in the control group (273 in the colon, 323 in the renal medulla, 298 in the adrenal gland, and 433 in the renal cortex). However, when exposed to osmotic stress (WR and GWR), ASEs decreased significantly under WR (*P* = 0.01, paired t-test) and GWR conditions (*P* = 0.03, paired t-test) (Figure 3d). Among the seven classical ASE types reported previously (Zhang et al., 2010), A3SS (alterative 3’ splicing site), A5SS (alterative 5’ splicing site) and retained introns contributed the most to the observed ASE reduction (Supplementary Figs. 6, 7, and 8). We did not observe any significant changes under SR, possibly because SR is less likely to induce strong osmotic stress. Reduction of ASEs in KRAB-ZFP genes will decrease KRAB-ZFP isoform numbers, facilitating the upregulation of their target genes or networks.

### Dominance of KRAB-ZFPs in network regulation

**Figure 4.**
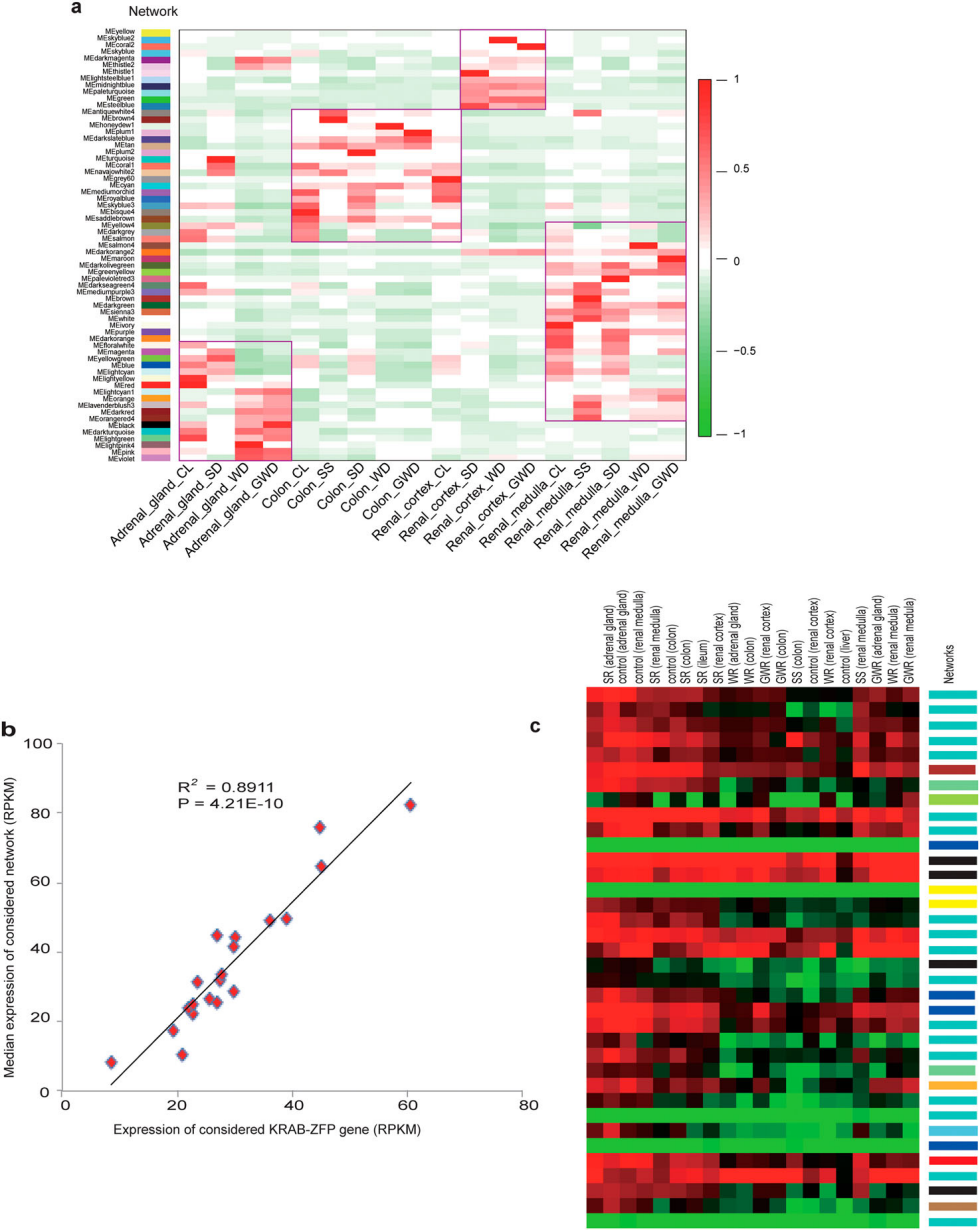
Network analysis in different samples and correlation between networks and KRAB-ZFP. (a) Network expression in different samples. Networks are labeled with colors indicated by the first color band. Other color bands on the right reveal highly correlated (red) or anti-correlated (blue) networks in specific samples. (b) Correlation between a hub KRAB-ZFP gene and its related network. The expression levels of the hub KRAB-ZFP gene/ The network's expression is represented by the median expression of the genes in this network. (c) Visualization of expression of all the tandem KRAB-ZFP genes in scaffold362. Scaffold362 contains the largest cluster of KRAB-ZFP genes in Bactrian camel. The network each gene involving in is listed on the color band on the right. Notes: (a) abbreviations labeled at bottom: CL is CG, SD is SR, WD is WR, GWD is GWR. (b) shows only one KRAB-ZFP gene. The *P*-value and R2 for each hub KRAB-ZFP gene is listed in Supplementary Table 11.

We next assessed whether KRAB-ZFPs play a dominant role in gene and network regulation under osmotic stress. A feasible and reasonable way to do this assessment was to see whether those genes were key members (hub genes) of the functional modules (hereafter referred to as networks) (Xue et al., 2013). We conducted weighted gene co-expression network analysis (WGCNA) (Langfelder & Horvath, 2008) on 20 transcriptome data sets to determine potential transcriptional networks. In total, we identified 65 potential networks (Figure 4a). Notably, 273 of 278 KRAB-ZFP genes expressed were involved in all of the networks. Given that those networks varied in function, the observation of different KRAB-ZFPs involving distinct networks demonstrated that these KRAB-ZFPs have functionally diverged. An example in scaffold 362 possessing 36 tandem KRAB-ZFP coding genes illustrates that even genes in the same cluster have diverged (Figure 4c). In addition, hub gene analysis showed that 110 KRAB-ZFPs were hub genes (kME ≥ 0.9), involved in more than half (n = 37) of those networks, indicating key roles of KRAB-ZFPs in these networks. To further confirm our conclusion, we plotted the expression of each hub KRAB-ZFP against that of its related networks among the 20 samples. The expression of a network is represented by the median expression among the genes in this network (Xue et al., 2013). As expected, we found a strong correlation (R2: 0.79–0.90, all *P* < 1E-7) between the two for all hub KRAB-ZFPs (Figure 4b and Supplementary Table 11). Thus, to the best of our knowledge, this is the first evidence that KRAB-ZFPs may be key regulators in those related networks.

### Transcriptional modules shift under osmotic stress

We next wanted to know whether these networks exhibited osmotic stressor specificity. To determine this, we visualized the expression changes of these networks in different tissues under the same treatment. If the performance (upregulated or downregulated) of one network was accordant in all tissues, then we would consider this network to be specific for this treatment. Nevertheless, none of these modules met our expectations. Instead, these networks exhibited tissue-specific patterns overall regardless of treatment (Figure 4a). To confirm our observation, we conducted a PCA on global gene expression; the results demonstrated that tissue specificity explained over 80% of the variation. Thus, we concluded that there is no special network responsible for osmotic stress; instead, tissue-specific expression patterns were consistent irrespective of treatment.

We were then interested in how these networks performed under different treatments within a specific tissue. First, we manually verified the renal medulla results and found that osmotic stress caused by different treatments resulted in expression changes of the networks compared to the control (Figure 4a), and different treatments gave rise to distinct expression change patterns. In the renal medulla, the blue network had the highest expression level in the control group. In contrast, this network was downregulated to a very low level in other treatments (Figure 4a). The yellow, steel-blue, and white networks had the highest expression under SS, WR, and GWR conditions, respectively (Supplementary Tables 12 and 13). The other three tissues also showed similar patterns. Since we had shown that KRAB-ZFPs may play a dominant role in network regulation, we suggest that network shift under osmotic stress may be driven by KRAB-ZFPs.

### Specific osmotic stress responses among tissues and among treatments

To obtain insight into the osmotic stress response mechanisms of different tissues under different treatments, we focused on the networks highly expressed under SS or WR treatments in varied tissues. We performed Gene Ontology (GO) and KEGG pathway enrichment analyses (*P* < 0.01, hypergeometric test) for each network to deduce its biological functions (Supplementary Tables 12 and 13). We first looked at the renal medulla and found that SS treatment induced a shift from ECM-receptor interaction, focal adhesion, or ribosome pathways (dark orange and ivory modules) to DNA or protein damage response-related pathways (mismatch repair, ABC transporters, cytosolic DNA-sensing pathway, endocytosis, heat shock protein binding, and unfolded protein binding), spliceosome and inositol phosphate metabolism pathways (white, brown, lavender blush 3, and orange-red 4 networks). The observations were consistent with the reports that osmotic stress could trigger DNA and protein damage in kidney cells (Bolen, 2001; Kültz & Chakravarty, 2001; Dmitrieva & Burg, 2007), or alterative splicing (van der Houven van Oordt et al., 2000). DNA damage signals can facilitate osmotic stress adaptation (Kültz, 2005). Upregulation of the inositol phosphate metabolism pathway under osmotic stress has been suggested to induce intracellular calcium waves, leading to reorganization of intracellular actin to prevent the cell volume from shrinking (Erickson & Guilak, 2000). Unlike SS treatment, WR induced the upregulation of biological functions related to cell growth regulation, insulin-like growth factor binding, and ferric iron binding (salmon 4 network). Insulin-like growth factors, e.g. IGF-1, can protect cells from hyperosmotic stress-induced apoptosis (Matthews & Feldman, 1996). As a consequence, the renal medulla has different mechanisms with which to address SS and WR treatments, although both give rise to osmotic stress.

In the colon, pathways including protein processing in the endoplasmic reticulum, amino sugar and nucleotide sugar metabolism, mucin-type o-glycan biosynthesis, glycosphingolipid biosynthesis, and the tachykinin receptor signaling pathway (medium orchid and bisque 4 networks) were highly expressed under CG conditions. However, they were substituted with arginine and proline metabolism, cytokine-cytokine receptor interaction, and salivary secretion pathway (antique white 4 and brown 4 networks) under SS conditions. An increase in the proline biosynthesis rate under osmotic stress has been reported in several studies (Boggess et al., 1976; Dandekar & Uratsu, 1988). Proline accumulation can augment intracellular osmotic pressure, thereby increasing the tolerance to osmotic stress. Intriguingly, this strategy has mostly been reported in plants and microorganisms. When treated with WR, the highly expressed pathways are the Gonadotropin-Releasing Hormone (GnRH) signaling pathway, those related to lactose biosynthesis, and gap junctions. The GnRH signaling pathway has been suggested to activate p38 Mitogen-Activated Protein Kinase (MAPK), an intracellular mediator of osmotic stress (Roberson et al., 1999). Hence, the GnRH signaling pathway may play a key role in the p38 MAPK-mediated osmotic stress response in the colon under WR treatment. Consequently, the colon coped with osmotic stress caused by SS and WR by different mechanisms.

Analyses of the renal cortex and adrenal gland also showed alternative response strategies under WR conditions. The renal cortex enhances the expression of biological functions associated with glutathione peroxidase activity and oxidoreductase activity (sky blue 2 network), both of which can defend cells against reactive oxygen species (Brooke et al., 2002; Koga et al., 2011). The adrenal gland responds to osmotic stress caused by WR treatment by overexpressing the terpenoid backbone biosynthesis pathway. Terpenoids are precursors of steroids and sterols (see URLs) that can maintain the stability of the cell membrane under osmotic stress (Dufourc, 2008; Dupont et al., 2011).

Overall, the mechanisms in the four tissues and different treatments are disparate, indicating the diversity of osmotic stress responses. Such diversity may arise from two aspects. (1) Heterogeneity of different tissues: Different tissues vary in many aspects, including the expression of functional modules or pathways and intracellular and intracellular osmolyte concentration, giving rise to distinct sensitivity to osmotic stress. (2) Varied osmotic pressure under different treatments: In our case, although both SS and WR induced osmotic stress, the osmotic pressure in space and time imposed by each was distinct even in the same tissue, leading to diverse response strategies.

We assessed the roles of KRAB-ZFPs in osmotic stress responses in vivo using transcriptome data from the adrenal gland, colon, renal cortex, and renal medulla under different treatments. For the first time, we concluded that KRAB-ZFPs possess a wide variety of regulatory roles in camels and drive transcriptional network shifts under osmotic stress. Our work provides insight into the genetic basis of the osmotic stress response.

## Materials and methods

### Sampling and sequencing

Fifteen healthy female Bactrian camels aged 6–9 years old were randomly divided into five groups. One group was used as a control (CG). The other four were treated with water restriction (WR), salt stress (SS), salt restriction (SR), or guzzling after water restriction (GWR). Each treatment lasted for four weeks (Supplementary Table 1). For the control group, salt, water, and hay were supplied as normal. Water was restricted in the WR group, and salt was restricted in the SR group. Regarding the SS group, each individual was fed with 200 grams on the first day, followed by 100 grams for three days. Treatments of camels in the GWR group were similar to WR, except that they guzzled water before being killed. All animal procedures were carried out according to the Inner Mongolia Association for Accreditation of Laboratory Animal Care, and the experimental protocols were approved by the Animal Care and Use Committee of Inner Mongolia Agricultural University. Blood and urine tests were conducted each week on all camels to monitor their physical conditions during the experiment.

For each group, six organs (liver, ileum, colon, renal cortex, renal medulla, and adrenal gland) were sampled. Total RNAs from the same treatment and the same tissue in a group were equally pooled. mRNA was isolated using magnetic beads with Oligo (dT) and used for library construction. Before sequencing, an Agilent 2100 Bioanalyzer and an ABI StepOnePlus Real-Time PCR System were used in quantification and qualification for each library. In this study, 20 of the 30 samples passed the QC step (adrenal gland and renal cortex for WR, GWR, SR, CG; colon and renal medulla for WR, GWR, SR, CG, SS; liver for CG; ileum for WR). Thus, these 20 samples were subjected to sequencing on a HiSeq2000 platform.

### Data generation

Blood and urine samples of each camel were collected each week for routine examination (Supplementary Tables 2–4). Since the data of the third week were incomplete, this week was excluded from further analysis. At the end of our experiment, the renal cortex, renal medulla, colon and adrenal gland, liver, and ileum were collected for mRNA extraction. Twenty of the 30 samples passed the quality control (adrenal gland and renal cortex for WR, GWR, SR, CG; colon and renal medulla for WR, GWR, SR, CG, SS; liver for CG; ileum for WR). Hence, the 20 samples were used for library construction with subsequent sequencing. In total, we produced 95 Gigabases (Gb) of transcriptome data for analysis (Supplementary Table 5). We mapped the reads of each sample to the published assembled genome of the Bactrian camel (Wu et al., 2015) using bowtie-0.12.7 (Langmead et al., 2009), with subsequently splice junctions (SJs) identification by Tophat-1.3.3 (Trapnell et al., 2009). We then identified alterative splicing types using in-house perl scripts. The reads per kilobase per million mapped reads (RPKM) value (Mortazavi et al., 2008) of each gene was calculated after aligning transcriptome reads to the published consensus gene set (Supplementary Fig. 9a).

### Gene expression calculation and alterative splicing detection

The Bactrian genome and gene set were downloaded from NCBI (accession number: JARL00000000). Transcriptome reads of each sample were mapped to the gene reference using SOAP2.20 (Li et al., 2009). Gene expression was measured as RPKM (Mortazavi et al., 2008) using in-house perl scripts. Potential splicing sites were identified using Tophat-1.3.3 (Trapnell et al., 2009) after aligning the transcriptome reads to the Bactrian reference genome using bowtie-0.12.7 (Langmead et al., 2009). Alterative splicing events were classified following previous reports.

### Differentially expressed genes identification

Differentially expressed genes (DEGs) analyses were performed between the control group and other treatments within the same tissue or among different tissues under the same treatment. The Poisson distribution was used to detect DEGs (Audic & Claverie, 1997). Genes with a false discovery rate (FDR) ≤ 0.01 and |log2(Fold change)| ≥ 1 were considered significantly upregulated or downregulated genes.

### Co-expression analysis

Co-expression analysis was performed using WGCNA, an R package for weighted correlation network analysis (Langfelder et al., 2008). To construct a signed weighted correlation network, we 1) generated a matrix of pairwise correlations between all pairs of genes across the 20 samples; 2) constructed an adjacency matrix by raising the co-expression measure to the power β = 12; 3) estimated the strength of two genes’ co-expression by calculating the topological overlap; 4) carried out average linkage hierarchical clustering to cluster the genes with highly similar co-expression relationships; and 5) defined functional modules by cutting the hierarchal clustering tree using the Dynamic Hybrid Tree Cut algorithm. Thus, a functional module could be considered a transcriptional gene network. The WGCNA package provides a means to evaluate the importance of a gene in a certain functional module, known as module eigengene-based connectivity (kME). Normally, when kME of a gene is ≥ 0.9, it is considered an intramodular hub gene (Xue et al., 2013).

### GO and KEGG analysis

To infer the function of each functional module, GO (Ashburner et al., 2000) and KEGG pathway (Kanehisa et al., 2000) enrichment analyses were conducted. The hypergeometric test was used to detect significantly enriched GO categories or KEGG pathways (*P* < 0.01) (Supplementary Fig. 9b).

## Data Availability

NCBI: BioSample accession: SAMN30996641; SubmissionID: SUB12093637.
